# A comparison of the effects of ibuprofen and rofecoxib on rabbit fibula osteotomy healing

**DOI:** 10.3109/17453670903316769

**Published:** 2009-10-01

**Authors:** J Patrick O'Connor, John T Capo, Virak Tan, Jessica A Cottrell, Michaele B Manigrasso, Nicholas Bontempo, J Russell Parsons

**Affiliations:** ^1^Department of Biochemistry and Molecular Biology and Department of Orthopaedics, University of Medicine and Dentistry of New Jersey, New Jersey Medical School and Graduate School of Biomedical SciencesNewark, NJUSA

## Abstract

**Background and purpose** Non-steroidal anti-inflammatory drugs (NSAIDs) inhibit cyclooxygenase (COX) activity, which is the rate-limiting enzyme in the synthesis of prostaglandins. Previous studies have indicated that NSAID therapy, and in particular NSAIDs that specifically target the inflammatory cyclooxygenase (COX-2), impair bone healing. We compared the effects of ibuprofen and rofecoxib on fibula osteotomy healing in rabbits to determine whether nominal, continuous inhibition of COX-2 with rofecoxib would differentially affect fracture healing more than cyclical inhibition of COX-2 using ibuprofen, which inhibits COX-1 and COX-2 and has a short half-life in vivo.

**Methods** Bilateral fibula osteotomies were done in 67 skeletally mature male New Zealand white rabbits. The rabbits were treated with placebo, rofecoxib (12.5 mg once a day), or ibuprofen (50 mg 3 times a day) for 28 days after surgery. Plasma ibuprofen levels were measured by HPLC analysis. Bone healing was assessed by histomorphometry at 3 and 6 weeks after osteotomy, and at 6 and 12 weeks by torsional mechanical testing.

**Results** Plasma ibuprofen levels peaked and declined between successive doses. Fracture callus morphology was abnormal in the rofecoxib-treated rabbits and torsional mechanical testing showed that fracture healing was impaired. Ibuprofen treatment caused persistence of cartilage within the fracture callus and reduced peak torque at 6 weeks after osteotomy as compared to the fibulas from the placebo-treated rabbits. In the specimens allowed to progress to possible healing, non-union was seen in 5 of the 26 fibulas from the rofecoxib-treated animals as compared to 1 of 24 in the placebo group and 1 of 30 in the ibuprofen treatment group.

**Interpretation** Continuous COX-2 inhibition as modeled by rofecoxib treatment appears to be more deleterious to fracture repair than cyclical cyclooxygenase inhibition as modeled by ibuprofen treatment. Ibuprofen treatment appeared to delay bone healing based upon the persistence of cartilage within the fracture callus and diminished shear modulus. Despite the ibuprofen-induced delay, rofecoxib treatment produced worse fracture (osteotomy) healing than ibuprofen treatment.

## Introduction

There is compelling experimental evidence that non-steroidal anti-inflammatory drug (NSAID) therapy impairs fracture healing in various animal models (O'Connor and Lysz 2008). Furthermore, NSAID impairment of fracture healing occurs by loss of cyclooxygenase-2 (COX-2) activity rather than cyclooxygenase-1 (COX-1) activity (Simon et al. 2002). Retrospective studies have also indicated that NSAID therapy can increase the incidence of fracture non-unions in humans (Burd et al. 2003). Prospective studies have shown that NSAID therapy is clinically useful for reducing the incidence and severity of heterotopic ossification (Burd et al. 2001). Thus, NSAID treatment has demonstrable effects on bone formation in animals as well as humans.

Despite the results of these studies, approximately 20% of patients treated for long bone fractures in emergency departments are prescribed NSAIDs to relieve inflammation and pain (Petrack et al. 1997). NSAIDs inhibit cyclooxygenase activity, thus reducing prostaglandin synthesis. In turn, reduced prostaglandin synthesis limits inflammation and the development of hyperalgesic pain (Steinmeyer 2000). Clearly, many of the fractures in patients treated with NSAIDs heal without sequelae. Several explanations could be suggested for why NSAID therapy is less deleterious for fracture healing in some patients than in others. Confounding co-morbidities such as age, diabetes, and smoking may have a significant additive effect that—when combined with NSAID treatment—severely compromises fracture healing.

Another variable that is likely to influence whether NSAID therapy impairs fracture healing is the NSAID itself. Duration of NSAID treatment and the fracture healing phase in which NSAIDs are used have been shown to substantially affect healing in an animal model (Simon and O'Connor 2007). In addition, which NSAID is used is likely to influence healing since the pharmacology and specificity of these compounds for the 2 different cyclooxygenases, COX-1 and COX-2, vary widely (Warner et al. 1999). We hypothesized that treatment with a traditional NSAID that has a short in vivo half-life (i.e. short-acting) will lead to daily periods when cyclooxygenase activity is not inhibited, and thus lead to a better fracture healing outcome. To test this hypothesis, we compared fracture healing using a rabbit fibula osteotomy model in animals treated with placebo, a short-acting, traditional NSAID (ibuprofen), or a long-acting, COX-2 selective NSAID (rofecoxib).

## Materials and methods

### Animal model

All animal procedures were approved by the New Jersey Medical School Institutional Animal Care and Use Committee. The study began with 67 skeletally mature, male New Zealand White (3.5-kg) rabbits (Table 1). The rabbits were acclimated for at least 1 week before surgery and were housed individually with a 12-h light-dark cycle and were given water and food ad libitum. 3 rabbits died from surgical anesthesia. 1 rabbit developed ischemia in its right forepaw following surgery that necessitated killing. 2 rabbits self-mutilated their right forepaw on day 5 post-surgery, which disqualified the animals from the study and these rabbits were killed. Animals were assigned to drug treatment groups based upon day of surgery. The surgeons were unaware of the assigned drug treatment group until after surgery was completed. Rabbits within a treatment group were randomly chosen for killing at prescribed time points and for histological or mechanical analysis.

**Table 1.** Disposition of the 67 rabbits used in this study

Treatment group	Total no.	Histology **^a^**		Mechanical testing **^b^**		Killed before endpoint			
		3 weeks	6 weeks	6 weeks	12 weeks	Anesthesia death	Infection	Ischemia	Self-mutilation
Placebo	21	3 (6)	3 (5)	6 (11;0;1)	6 (9;1;2)	1	2	–	–
Ibuprofen	25	3 (6)	3 (5)	7 (12;1;1)	8 (12;0;4)	–	1	1	2
Rofecoxib	21	3 (6)	3 (6)	6 (10;2;0)	7 (11;3;0)	2	–	–	–
**^a^** The number of fibulas tested for each group is given in parentheses.										**^b^** The disposition of the fibulas for each group is indicated in parentheses (number tested; number of non-unions; number not used for other reasons).

Practice surgeries were performed on 5 separate rabbits that had been used to determine an effective ibuprofen drug dose, and they were not included in the total number of animals in the study (Table 1). All procedures described below were applied to the practice surgery rabbits, except that they were killed at 2 or 4 weeks after surgery. The practice rabbits were not treated with ibuprofen or rofecoxib following surgery. Radiographs of the fibula osteotomies demonstrated that the fibulas had not bridged with new bone after 4 weeks of healing. Mechanical testing of fibula osteotomies would be uninformative if the osteotomies in the control rabbits (with no drug treatment) had not bridged with bone. Based upon these observations, we decided to collect specimens at 6 and 12 weeks for mechanical testing and 3 and 6 weeks for histological analysis.

### Surgical procedure

Rabbits were fasted overnight before surgery. Each rabbit was medicated with glycopyrrolate (0.01 mg/kg) and acepromazine (0.75 mg/kg) as a sedative by intramuscular (IM) injection. After 15 min, anesthesia was induced with ketamine (55 mg/kg) and xylazine (5 mg/kg) by IM injection. The rabbit was administered morphine (1 mg/kg) for immediate postoperative pain relief and enrofloxacin (10 mg/kg) as a prophylactic antibiotic, both by IM injection. The posterior-lateral aspect of each hind limb was shaved from below the knee to the ankle. The back was shaved above the scapula and one half of a 25-μg-per-hour fentanyl patch was applied to this site with sutures through the skin, for postoperative pain control. The anesthetized rabbit was moved to the surgical table and placed supine on a heated pad, and the surgical sites were prepared and draped in a standard fashion. Before surgery, each surgical site was injected with 0.5–1 mL of 0.25% bupivacaine for pain control.

A 4-cm incision was made on the lateral aspect of each hind limb at the mid-diaphyseal level of the fibula. The diaphysis of the fibula was exposed by blunt dissection of the overlying muscle and a 1.5-mm-wide osteotomy was created using a compressed gas-driven mini-driver with a reciprocating saw blade. The wound was irrigated with saline and then closed in layers using interrupted, resorbable sutures. It was treated with an ointment containing polymyxin B, bacitracin, and neomycin (Triple Antibiotic Ointment; Taro Pharmaceuticals USA, Inc., Hawthorne, NY). Postoperative radiographs were used to confirm the quality of the osteotomy. For a separate experiment, the flexor tendons in the right forepaw of each animal were transected and repaired. The skin was closed at all incisions with interrupted resorbable sutures and only the right forelimb was immobilized with a below-elbow splint. The hind-limbs and left forelimb were not immobilized, and were permitted free motion.

Following surgery, the rabbits were allowed to recover in heated incubators until the animals were upright, alert, and had reached a normal body temperature. They were then returned to their cages. Morphine (1 mg/kg IM) was administered 4 and 8 h post-procedure. An additional dose of enrofloxacin (5 mg/kg IM) was administered before returning the rabbits to their cages after surgery, and then twice a day for the 3 days following surgery. The rabbits were permitted free weight bearing and unrestricted use of their limbs. The right forepaw splint was removed at 14 days. Postoperative, superficial, surgical skin wound infections were treated by application of an antibiotic ointment, or with intramuscular injections of enrofloxacin (10 mg/kg) as directed by the veterinary staff, until the infection was resolved. The animals were killed by intravenous injection of pentobarbital (390 mg) and phenytoin (50 mg).

### Drugs and dosing

A pilot study was conducted to identify a suitable ibuprofen dose. Rabbits were given an oral dose of ibuprofen and peripheral blood was collected from the ear vein for analysis of COX-2 activity as an indirect measure of ibuprofen effect. COX-2 activity was measured by determining prostaglandin E2 levels in blood sera after lipopolysaccharide stimulation, as described previously (Brideau et al. 1996). We found that 25-, 50-, and 100-mg doses of ibuprofen per rabbit (approximately 7.5, 15, and 30 mg/kg) inhibited COX-2 activity 1 h after oral administration. Subsequent experiments using a 50 mg dose of ibuprofen per rabbit showed that maximal COX-2 inhibition occurred between 2 and 4 h after dosing. This was consistent with previous studies showing that peak ibuprofen plasma levels occur between 1.5 and 3 h after oral administration, that elimination time is dependent upon dose, and that elimination half-life is very short: less than 1 h for intravenously administered ibuprofen (Williams et al. 1991). Thus, we chose to use a 50-mg oral dose administered 3 times a day, at least 4 h apart (150 mg/day). This approximates the recommended veterinary dose of 7.5 mg/kg given 4 times a day, which would equal approximately 100 mg of ibuprofen per day for a 3.5-kg rabbit. The rofecoxib dose of 12.5 mg per rabbit (3.5 mg/kg) was based upon a previous study that showed that this dose of rofecoxib given orally as a single daily dose impaired bone formation in a rabbit model (Goodman et al. 2002).

**Figure 1.**
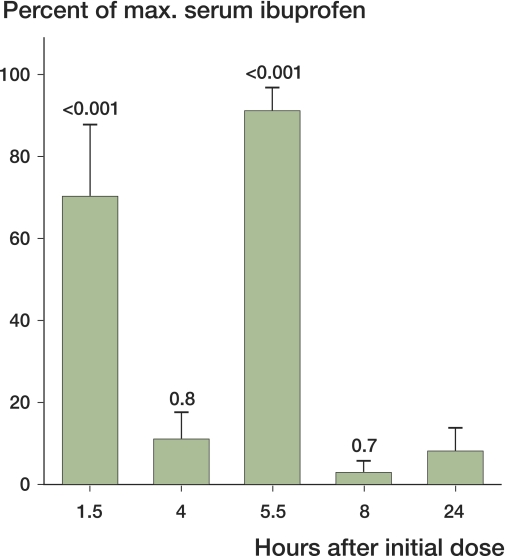
Variation in plasma ibuprofen levels. Plasma ibuprofen levels were statistically elevated 1.5 h after the 8 a.m. and noon doses, but had declined by 4 h after dosing. Data were compared to the 24-h sample values (16 h after the 4 p.m. dose) and the p-values are indicated.

Plasma ibuprofen levels were measured using a previously described HPLC-based assay to confirm that plasma ibuprofen levels varied between doses (Teng et al. 2003). On day 4 after surgery, blood was collected into heparin from the ear vein of 4 ibuprofen-treated rabbits at 1.5, 4, 5.5, 8, and 24 h after ibuprofen (50 mg) administration at 0, 4, and 8 h. Plasma was separated by centrifugation and stored at –20°C. Aliquots of plasma were thawed, spiked with a naproxen internal standard, and the serum proteins were precipitated as described previously (Teng et al. 2003). The resulting supernatant containing the ibuprofen was dried, dissolved in 70% methanol, and applied to a C18 reverse-phase column (Zorbax Eclipse XDB-C18 5-μm, 4.6 × 150 mm; Aligent, Santa Clara, CA). The column was developed with 40% water (the pH was adjusted to 2.6 with phosphoric acid) and 60% acetonitrile at a flow-rate of 2 mL/min and column temperature of 40°C using a Dionex GP40 chromatographic HPLC system (Sunnyvale, CA). Ibuprofen was detected by absorbance at 220 nm and quantified using a standard curve of (±)-ibuprofen (Cayman Chemical Co., Ann Arbor, MI). Data from different rabbits were normalized as the percentage of the maximum level, which occurred at either 1.5 or 5.5 h in all rabbits (Figure 1).

Ibuprofen, rofecoxib, and placebo were given orally to the rabbits via chewable tablets. For ibuprofen treatment, the rabbits were given half of a Junior-Strength Advil Chewable Tablet (100 mg ibuprofen per tablet; Wyeth Consumer Healthcare, Richmond, VA) 3 times a day. For rofecoxib treatment, the rabbits were given one quarter of a custom-made chewable tablet (50 mg rofecoxib per tablet) in the morning and then one quarter of a chewable placebo tablet (Custom MDs, Bio-Serv, Frenchtown, NJ) for the remaining doses. The rofecoxib chewable tablets were prepared by Bio-Serv from VIOXX tablets (Merck, Whitehouse Station, NJ). The placebo-treated rabbits were given one quarter of a chewable placebo tablet 3 times a day. Animals were dosed 3 times a day: in the morning (8–9 a.m.), at noon (12–1 p.m.), and in the late afternoon (4–6 p.m.). The first drug dose was delivered on the morning after surgery. Drugs were administered for 28 days after surgery.

Rabbits were randomized for drug treatment by date of surgery. The surgeons were unaware of the drug treatment regime that the rabbits were to undergo following surgery. Rabbits within a treatment group were randomly chosen for killing, and for histological or mechanical analysis. All histomorphometric and mechanical testing procedures were performed using rabbit identification numbers only, without identification as to which drug treatment group or time point the rabbit belonged.

### Histology

Rabbits were killed at 3 and 6 weeks after surgery for histological analysis. Fibulas were collected, radiographed, fixed in formalin, and embedded in polymethylmethacrylate using a previously described technique (Baron et al. 1983). At least one longitudinal section through the osteotomy site was cut from each fibula and stained with van Gieson's picrofuchsin and Stevenel's blue to determine the extent of bone healing (Maniatopoulos et al. 1986). Digital images of each section were captured using a camera and microscope. Histomorphometric measurements of total callus, new bone, and cartilage area were made using Image Pro version 5 software (Media Cybernetics, Bethesda, MD). The area of each tissue type was determined and percentages calculated. Two histological specimens were lost from the study because of preparation errors (Table 1). Values for fibulas obtained from the same animal were averaged and the mean value was used for subsequent calculations and comparisons.

### Mechanical testing of fibulas

Fibula samples were collected at 6 and 12 weeks after surgery, radiographed, and stored at –20°C before mechanical analysis. Osteotomy external callus dimensions were measured with a digital caliper prior to testing. The fibulas were potted in one-inch hex-nuts using Wood's metal, the gauge length recorded, and then each bone was tested to failure in torsion using an MTS servo-hydraulic test machine (Eden Prairie, MN) with a 20-Nm reaction torque cell at a rate of 2 degrees per second. Force and angular displacement were recorded every 0.05 seconds. Wall thickness was measured from 6 fibula histological specimens described above, and the mean value was used for all calculations. Torsional rigidity, polar moment of inertia, peak torque, maximum shear stress, and shear modulus were calculated as described previously (Simon et al. 2002). Values for fibulas obtained from the same animal were averaged and the mean value was used for subsequent calculations and comparisons.

Of the 80 fibulas that were available for mechanical testing, 15 were lost from the study for different reasons (Table 1). 2 fibulas were lost during harvest by inadvertent breaking before testing, 1 in the 6-week placebo group and 1 in the 6-week ibuprofen group. One fibula in the 12-week ibuprofen group could not be tested because the osteotomy was too distal in the bone. Three of the fibulas from the 12-week ibuprofen treatment group and 2 fibulas from the 12-week placebo group did not fail at or through the fracture site during testing, and were disqualified. We suspect that failure at a site other than the osteotomy callus indicates that a stress riser may have been created during preparation, which led to failure at a non-osteotomy site. One fibula from the 12-week placebo group, 1 fibula from the 6-week ibuprofen group, 2 fibulas from the 6-week rofecoxib group, and 3 fibulas from the 12-week rofecoxib group were not tested because of non-unions.

### Group sizes and statistics

Differences between treatment groups at each time point were assessed by analysis of variance (ANOVA). Since both fibulas from the same animal were tested, the outcome parameter values for both fibulas were averaged and the average value was used for any subsequent analyses. For the 3-week histomorphometry analysis, 3 treatment groups were compared using values from 9 rabbits. For the 6-week histomorphometry analysis, 3 treatment groups were compared using values from 9 rabbits. For the 6-week mechanical testing analysis, 3 treatment groups were compared using values from 19 rabbits. For the 12-week mechanical analysis, 3 treatment groups were compared using values from 19 rabbits. For all ANOVA analyses, drug treatment was used as the independent variable and the measured outcome parameter was used as the dependent variable. If the overall treatment effect was significant (p < 0.05), between-treatment comparisons were performed using F-tests. Plasma ibuprofen values were compared to the 24-h values using ANOVA and post-hoc Holm-Sidak tests. All statistical analyses were performed using SigmaStat version 3 software.

## Results

### Plasma ibuprofen levels

An HPLC analysis of plasma ibuprofen confirmed preliminary experiments and previous reports that ibuprofen is quickly eliminated in rabbits (Williams et al. 1991). Rabbits were given 50-mg oral doses of ibuprofen at 8 a.m. (zero time) on day 4 after surgery and then at noon (4 h after zero time) and at 4 p.m. (8 h after zero time). Blood was collected at 1.5, 4, 5.5, 8, and 24 h after the initial dose on day 4. Plasma levels of ibuprofen were significantly elevated 1.5 h after an oral 50-mg dose of ibuprofen (1.5- and 5.5-h time points). By 4 h after administration (4- and 8-h time points), plasma ibuprofen declined to levels that were no different from those in the 24-h samples. The mean maximum serum ibuprofen level was 6.7 μg/mL (SEM 3.3, n = 4).

### Histology and radiography

**Figure 2.**
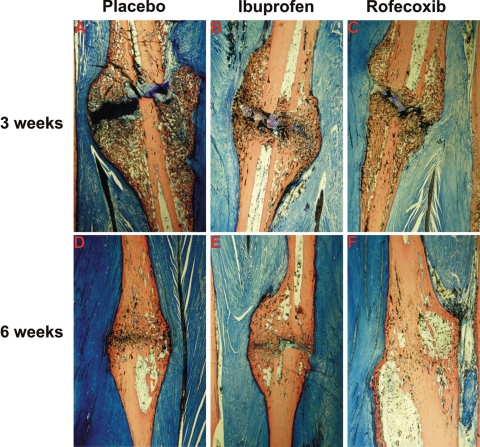
Representative histological sections through the fibula osteotomy callus. Panels A–C show sections from rabbit fibulas after 3 weeks of healing that were treated with placebo, ibuprofen, or rofecoxib as indicated. Similarly, panels D–F show sections from rabbit fibulas after 6 weeks of healing. Note the shell-like morphology of the osteotomy callus in the rofecoxib-treated rabbit after 6 weeks of healing.

**Figure 3.**
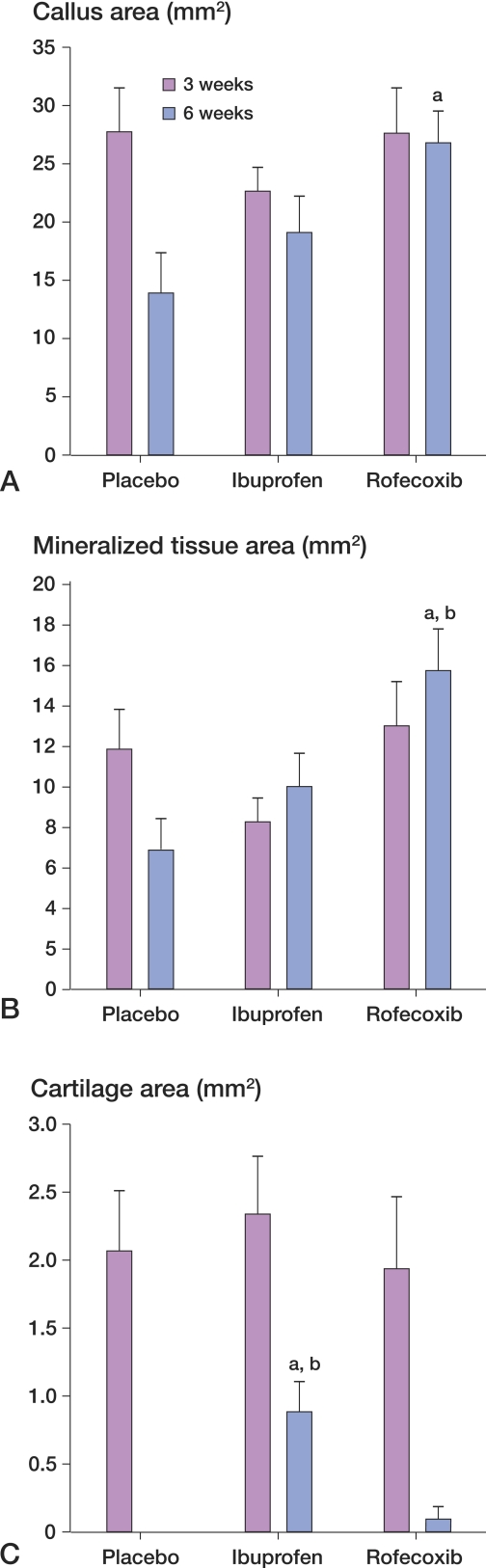
Histomorphometric analysis of the effect of NSAIDs on fracture repair. Shown are the mean and standard error after 3 weeks (red bars) and 6 weeks (blue bars) of healing for callus area (panel A), mineralized tissue area (panel B), and cartilage area (panel C). No statistical differences were found after 3 weeks of healing. Mean values that are statistically significantly different from the placebo value are indicated with an “a”. Mean values that are statistically different between the ibuprofen and rofecoxib treatment groups are indicated with “b”.

Healing of the rabbit fibula osteotomies was measured after 3 and 6 weeks by histomorphometry (Figure 2). Longitudinal sections parallel to the plane formed by the long axes of the tibia and fibula were measured to determine mineralized tissue (bone and mineralized cartilage) and cartilaginous tissue areas. There were no significant differences in callus area, mineralized tissue area, or cartilage area between the placebo, ibuprofen, and rofecoxib treatment groups after 3 weeks of healing. In contrast, at 6 weeks of healing there was significantly more cartilage in the osteotomy callus of the ibuprofen-treated rabbits than in the placebo- or rofecoxib-treated rabbits (Table 2 and Figure 3). In addition, there was more overall mineralized tissue and callus area in the rabbit fibula osteotomy calluses of the rofecoxib treatment group than in the ibuprofen or placebo treatment group.

**Table 2.** Summary of fibula histomorphometric analysis

	Group size	Mean (SD)	p-value			Difference in means (95% CI)	
			ANOVA	(vs. placebo)	(ibu. vs. rof.)	(vs. placebo)	(ibu. vs. rof.)
3 weeks post-fracture							
Callus size (mm^2^)			0.7				
Placebo	3	27.7 (9.7)					
Ibuprofen	3	22.6 (2.8)		–		5.1 (-11.2 to 21.3)	
Rofecoxib	3	27.6 (9.0)		–	–	0.1 (-21.2 to 21.4)	5.0 (-20.2 to 10.2)
Mineralized tissue (mm^2^)			0.4				
Placebo	3	11.9 (4.5)					
Ibuprofen	3	8.3 (1.6)		–		3.6 (-4.1 to 11.3)	
Rofecoxib	3	13.0 (5.3)		–	–	-1.2 (-12.3 to 10.0)	-4.7 (-13.6 to 4.2)
Cartilage (mm^2^)			0.9				
Placebo	3	2.1 (0.8)					
Ibuprofen	3	2.3 (0.9)		–		-0.3 (-2.2 to 1.6)	
Rofecoxib	3	1.9 (1.1)		–	–	-0.1 (-2.1 to 2.4)	0.4 (-1.9 to 2.7)
6 weeks post-fracture							
Callus size (mm^2^)			< 0.05				
Placebo	3	14.7 (3.6)					
Ibuprofen	3	17.7 (6.1)		0.5		-3.0 (-14.3 to 8.3)	
Rofecoxib	3	26.8 (4.2)		0.02	0.06	-12.1 (-20.8 to -3.3)	-9.1 (-20.9 to 2.8)
Mineralized tissue (mm^2^)			< 0.02				
Placebo	3	7.2 (1.6)					
Ibuprofen	3	9.2 (3.4)		0.4		-2.0 (-8.1 to 4.1)	
Rofecoxib	3	15.7 (2.5)		0.007	0.02	-8.5 (-13.210 to -3.781)	-6.5 (-13.3 to 0.3)
Cartilage (mm^2^)			0.002				
Placebo	3	0 (0)					
Ibuprofen	3	0.9 (0.3)		0.001		-0.9 (-1.4 to -0.4)	
Rofecoxib	3	0.1 (0.2)		0.6	0.002	-0.1 (-0.3 to 0.2)	0.8 (0.3 to 1.4)

**Figure 4.**
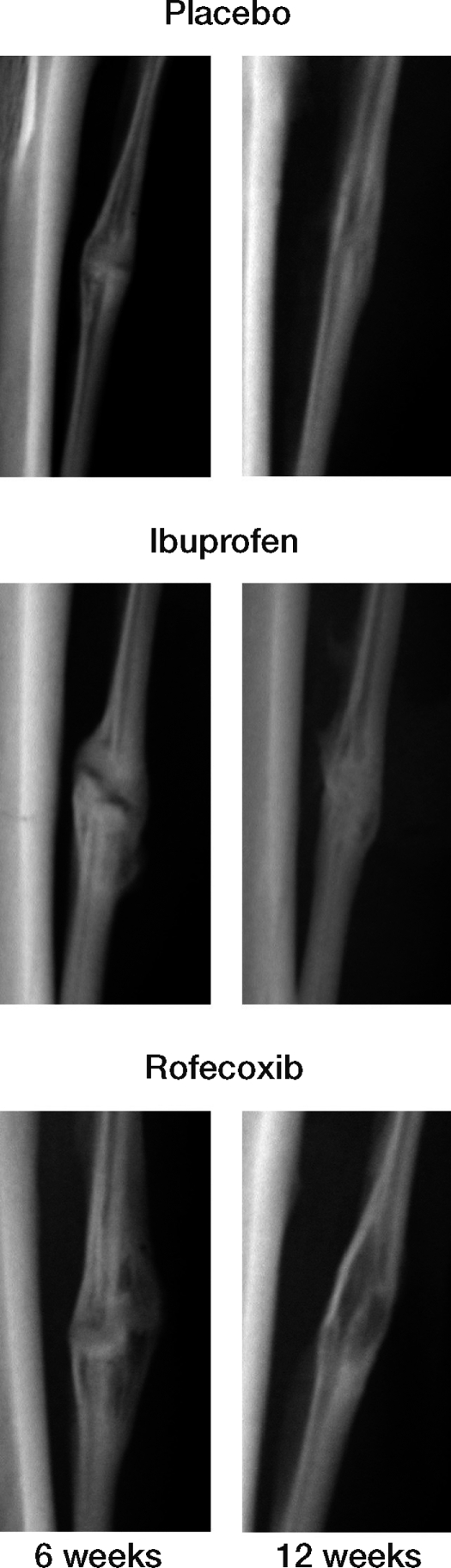
Representative images of the fibula osteotomy calluses: lateral radiographs made from the resected lower hind limbs of each rabbit at 6 and 12 weeks after osteotomy. In each radiograph, the tibia cortex can be seen on the left margin and each is oriented with the proximal end up. Note the persistence of the osteotomy gap after 6 weeks in the ibuprofen-treated rabbit, suggesting a delay in healing. After 12 weeks, the osteotomies in the placebo- and ibuprofen-treated rabbits appear to have healed well. In contrast, significant radiolucent areas are evident in the 6- and 12-week calluses of the rofecoxib-treated rabbits.

Bone bridging of the osteotomy gap was evident in most of the fibulas (Figures 2 and 4). After 12 weeks of healing, the fibulas from the ibuprofen-treated rabbits appeared to be bridged with new bone and appeared to have undergone considerable remodeling, as callus size appeared smaller (Table 2 and Figures 2–4). Similar observations were made in most of the rofecoxib-treated rabbits except that large areas of the callus appeared to be poorly mineralized (Figures 2 and 4).

### Mechanical testing

7 fibulas were observed to be non-unions and thus could not undergo mechanical testing (Table 1). There was 1 non-union in the placebo group, 1 non-union in the ibuprofen group, and there were 5 non-unions in the rofecoxib group. Chi-square test indicated that the larger proportion of non-unions in the rofecoxib group was not significant (p = 0.1). The fibulas with a non-union could not be used for the mechanical testing; thus, the torsional mechanical testing data only reflect results from fibulas in which the osteotomy was bridged with bone.

The fibular osteotomy sites were measured by torsional mechanical testing after 6 and 12 weeks of healing (Table 3). Rofecoxib treatment significantly reduced the material properties (peak torque and maximum rigidity) and shear modulus of the callus after 6 weeks of healing as compared to the placebo-treated rabbits. After 12 weeks of healing, no difference was detected between the structural properties of the rofecoxib-treated and placebo-treated rabbits, but the material properties (maximum shear stress and shear modulus) were significantly less. Similarly to the placebo comparison, the material properties of the calluses from the rofecoxib-treated rabbits were significantly less than those from the ibuprofen-treated rabbits after twelve weeks of healing. No differences in the mechanical properties of callus were detected between the ibuprofen-treated rabbits and the placebo-treated rabbits at either time point.

**Table 3.** Summary of fibula torsional mechanical testing analysis

	Group size	Mean (SD)	p-values			Difference in means (95% CI)	
			ANOVA	(vs. placebo)	(ibu. vs. rof.)	(vs. placebo)	(ibu. vs. rof.)
6 weeks post-fracture							
Peak torque (Nmm)			0.02				
Placebo	6	227 (38)					
Ibuprofen	7	181 (43)		0.04		47 (-3 to 96)	
Rofecoxib	6	165 (26)		0.01	0.5	62 (21 to 104)	16 (-29 to 60)
Max. rigidity (Nmm^2^/rad)			0.02				
Placebo	6	7,452 (2,300)					
Ibuprofen	7	6,175 (1,813)		0.2		1,277 (-1,232 to 3,785)	
Rofecoxib	6	4,304 (668)		0.006	0.07	3,147 (969 to 5,326)	1,870 (141 to 3,600)
Max. shear stress (MPa)			0.2				
Placebo	6	36 (8)					
Ibuprofen	7	33 (15)		–		3.2 (-11.9 to 18.4)	
Rofecoxib	6	24 (8)		–	–	12.1 (2.1 to 22.1)	8.9 (-6.1 to 23.8)
Shear modulus (MPa)			< 0.05				
Placebo	6	433 (177)					
Ibuprofen	7	348 (114)		0.3		86 (-93 to 264)	
Rofecoxib	6	231 (83)		0.02	0.1	202 (24 to 380)	117 (-7 to 240)
12 weeks post-fracture							
Peak torque (Nmm)			0.3				
Placebo	5	210 (58)					
Ibuprofen	7	250 (101)		–		-40 (-154 to 73)	
Rofecoxib	7	185 (47)		–	–	25 (-43 to 92)	65 (-27 to 157)
Max. rigidity (Nmm^2^/rad)			0.2				
Placebo	5	8,917 (2,917)					
Ibuprofen	7	8,690 (4,698)		–		227 (-5,096 to 5,550)	
Rofecoxib	7	5,766 (1,572)		–	–	3,151 (267 to 6,035)	2,924 (-1,156 to 7,004)
Max. shear stress (MPa)			0.04				
Placebo	5	51 (17)					
Ibuprofen	7	53 (22)		0.9		-1.2 (-27.8 to 25.3)	
Rofecoxib	7	30 (8)		0.04	0.02	21.8 (5.8 to 37.8)	23.0 (3.4 to 42.7)
Shear modulus (MPa)			< 0.02				
Placebo	5	894 (373)					
Ibuprofen	7	711 (386)		0.3		184 (-313 to 681)	
Rofecoxib	7	338 (116)		0.007	0.04	556 (227 to 886)	373 (41 to 705)

## Discussion

As expected from previous studies, rofecoxib treatment impaired healing of the rabbit fibula osteotomies (Goodman et al. 2002, Simon et al. 2002). Rofecoxib treatment initially led to decreases in the 6-week osteotomy callus structural and material properties as compared to the placebo-treated rabbits (Table 3). 12 weeks after surgery, however, rofecoxib treatment only led to decreases in callus material properties as compared to the placebo or ibuprofen-treated rabbits. The similarity in callus structural properties between the rofecoxib-treated and the placebo or ibuprofen-treated rabbits probably relates to the larger size of the calluses in the rofecoxib-treated rabbits (Table 2), which would provide a substantial structural advantage despite the apparent poor quality of the bone—as reflected by its histological and radiographic appearance (Figures 2 and 4) and material properties (Table 3). While rofecoxib treatment did not ultimately prohibit fracture repair in this rabbit study, it was clearly detrimental. 5 of the 26 fibulas from the rofecoxib-treated rabbits resulted in non-unions as compared to 1 of 24 in the placebo group and 1 of 30 in the ibuprofen treatment group. One can assume that the non-unions would have had very poor mechanical properties; thus, our dataset positively skews the mechanical testing results from the rofecoxib treatment group, since non-unions were not tested. Consequently, it is likely that we have underestimated the negative mechanical effects of rofecoxib treatment on fracture repair in this animal model.

In contrast, ibuprofen treatment delayed healing of the rabbit fibula osteotomies—which was evident from the substantial amount of cartilage remaining in the osteotomy callus and the diminished peak torque after 6 weeks of healing (Tables 2 and 3). This delay had no apparent lasting effect, however, since no differences in the mechanical properties of callus were detected between the placebo and ibuprofen treatment groups after 12 weeks of healing.

These data support our hypothesis that short-acting NSAID therapy is less deleterious to fracture healing than long-acting NSAID therapy. A shortcoming of our study is that no experiments were performed to verify plasma levels of rofecoxib in the treated rabbits. However, an analysis of plasma ibuprofen levels (Figure 1) confirmed our preliminary experiments indicating that a single oral dose of 50 mg ibuprofen provided significant COX-2 inhibition for approximately 4 h (between 1 and 5 h after dosing; data not shown). Thus, we suspect that the ibuprofen dosing regimen used allowed consistent inhibition of COX-2 for no more than 14 h each day. This would leave a substantial period of at least 10 h when COX-2 function was not impaired.

Unfortunately, the pharmacokinetics of rofecoxib in rabbits has not been described in the literature. In male rats, the plasma concentration of rofecoxib peaks 0.5 h after administration and has a half-life of 4.3 to 6 h, depending upon the dose administered (Halpin et al. 2000). Assuming the pharmacokinetics of rofecoxib are similar between rats and rabbits, then the dose of rofecoxib (approximately 3.5 mg/kg) administered to the rabbits should have provided substantial COX-2 inhibition over most of the 24-h dosing cycle. Thus, it would appear that during fracture healing, daily periods when COX-2 activity is not inhibited lead to better healing outcomes than when COX-2 activity is continuously inhibited.

The effects of NSAID treatment on bone healing has been extensively studied in animal models (O'Connor and Lysz 2008). While most of these studies have concluded that NSAID therapy impairs fracture healing, the severity of impairment has varied extensively between studies. In our earlier work, we found that celecoxib therapy (4 mg/kg per day) appeared to impair fracture healing in male rats based upon histological and radiographic observations, yet torsional mechanical testing data failed to show any significant difference from control specimens after 8 weeks of healing (Simon et al. 2002). In a more recent study, we found that celecoxib therapy (4 mg/kg per day) significantly impaired fracture healing in female rats, including torsional mechanical testing data after 8 weeks of healing (Simon and O'Connor 2007). A critical difference between these studies was the use of male and female rats and the dramatic difference in celecoxib elimination times between the two sexes. The elimination half-life of celecoxib in female rats is 14 h, which is similar to that in humans (Paulson et al. 2000). In contrast, the elimination half-life of celecoxib in male rats is approximately 4 h. Thus, a single 4 mg/kg celecoxib dose can inhibit COX-2 over a 24-h time period in female rats but not in male rats.

Based upon the present experimental findings, the difference in fracture healing outcomes measured between male and female rats treated with celecoxib most likely reflects the periods each day when COX-2 activity was not inhibited in the male rats. Similarly, studies that examined the effects of aspirin, indomethacin, ibuprofen, or other short-acting NSAIDs on fracture healing have shown a similar pattern of impaired healing characterized by a delay rather than complete inhibition of healing (Allen et al. 1980, Altman et al. 1995).

Together, these animal-based studies suggest that fracture patients should avoid using NSAIDs to control fracture pain and inflammation. However, if NSAIDs are used, a short-acting NSAID appears to be preferable to using a long-acting NSAID. It should be stressed that patients may use large doses or more frequent doses of a short-acting NSAID that could extend the daily period in which COX-2 is inhibited, and thus still potentially delay healing.

The mechanism by which NSAIDs impair fracture healing remains unknown. Clearly, NSAIDs inhibit cyclooxygenase activity and reduce fracture site prostaglandin levels (Simon and O'Connor 2007). However, the question of which prostaglandins are critical for healing, when each is required, from which cells the prostaglandin are made, and which cells respond to the prostaglandins is not clear. Infusion of large doses of prostaglandin E2 or prostaglandin E2 receptor agonists can promote healing (Keller et al. 1993, Yoshida et al. 2002, Paralkar et al. 2003). However, it is unclear—given the large doses of prostaglandin E2 or receptor agonists—that activation of other prostaglandin receptors did not occur or that the treatment itself did not induce COX-2 activity, leading to an indirect activation of other prostaglandin receptors. Angiogenesis is necessary for fracture healing, and prostaglandins can induce angiogenesis (Hausman et al. 2001, Seno et al. 2002). Accordingly, NSAID therapy has been associated with reduced fracture callus blood flow (Murnaghan et al. 2006). Whether the NSAID therapy directly impairs angiogenesis during fracture healing or whether persistence of avascular cartilage in the callus reflects the reduced blood flow still remains to be determined.

To summarize, we have presented data supporting the hypothesis that short-acting NSAID therapy (ibuprofen) is less deleterious to fracture healing than long-acting NSAIDs (rofecoxib). Furthermore, we suggest that this difference may be due to daily periods when COX-2 is active in animals treated with short-acting NSAIDs. These data may help to resolve variations in results found between different animal studies that have examined the effects of NSAIDs on fracture healing and variations in the clinical experience of many physicians and patients who have prescribed or used NSAIDs to control pain and inflammation following a fracture.
